# High-Resolution Copy Number Patterns From Clinically Relevant FFPE Material

**DOI:** 10.1038/s41598-019-45210-2

**Published:** 2019-06-20

**Authors:** Anastasia Filia, Alastair Droop, Mark Harland, Helene Thygesen, Juliette Randerson-Moor, Helen Snowden, Claire Taylor, Joey Mark S. Diaz, Joanna Pozniak, Jérémie Nsengimana, Jon Laye, Julia A. Newton-Bishop, D. Timothy Bishop

**Affiliations:** 1 Section of Epidemiology and Biostatistics, Leeds Institute of Medical Research at St James’s, University of Leeds, Leeds, United Kingdom; 2 Centre for Translational Research, Biomedical Research Foundation of the Academy of Athens (BRFAA), Athens, Greece; 3 MRC Medical Bioinformatics Centre, Leeds Institute of Data Analytics, University of Leeds, Leeds, United Kingdom

**Keywords:** Melanoma, Molecular medicine, Cancer

## Abstract

Systematic tumour profiling is essential for biomarker research and clinically for assessing response to therapy. Solving the challenge of delivering informative copy number (CN) profiles from formalin-fixed paraffin embedded (FFPE) material, the only likely readily available biospecimen for most cancers, involves successful processing of small quantities of degraded DNA. To investigate the potential for analysis of such lesions, whole-genome CNVseq was applied to 300 FFPE primary tumour samples, obtained from a large-scale epidemiological study of melanoma. The quality and the discriminatory power of CNVseq was assessed. Libraries were successfully generated for 93% of blocks, with input DNA quantity being the only predictor of success (success rate dropped to 65% if <20 ng available); 3% of libraries were dropped because of low sequence alignment rates. Technical replicates showed high reproducibility. Comparison with targeted CN assessment showed consistency with the Next Generation Sequencing (NGS) analysis. We were able to detect and distinguish CN changes with a resolution of ≤10 kb. To demonstrate performance, we report the spectrum of genomic CN alterations (CNAs) detected at 9p21, the major site of CN change in melanoma. This successful analysis of CN in FFPE material using NGS provides proof of principle for intensive examination of population-based samples.

## Introduction

The documentation of small regions of genomic copy number alterations (CNAs) in tumours is now a standard component of characterising the genomic structure of a neoplasm^1^. A variety of techniques exist for doing this, with reliable and reproducible findings overall when either the DNA is largely intact (as from fresh-frozen samples) or there are significant quantities of starting material available. Difficulties arise when the starting material is degraded or limited. Malignant melanoma represents such a challenge: most lesions removed are small, of the order of 1–2 mm in depth, and require histopathological examination, so the entire excised melanoma tumour is routinely formalin-fixed and paraffin wax embedded (FFPE) for diagnosis, precluding retaining frozen tissue for research.

Available techniques for genomic analysis of FFPE material include array-based methods and whole genome amplification of the FFPE DNA prior to analysis^2^. Genome-wide single nucleotide polymorphism (SNP) arrays, such as the OncoScan^®^ FFPE array^3^, are becoming more feasible, especially when specifically constructed for coverage and content, and have shown good reliability and reproducibility. These arrays have the potential to work well when particularly focused on specific regions (but hence are less informative for discovery). The OncoScan^®^ array offers 50- to 100-kb resolution for 900 cancer-related genes, and 300-kb resolution in other areas. However, these arrays still require about 80 ng of DNA^3^. Whole genome amplification performance degrades with fragmented DNA and there are concerns that biased amplification can be erroneously detected as a CNA^2^.

Detailed analysis of cell lines and large fresh frozen melanoma primary tissue has identified consistent CNAs, including distinct patterns of copy number (CN) changes associated with *BRAF* versus *NRAS* mutant melanomas^4^ and chromothripsis associated with patient prognosis for FFPE primary melanoma with a median thickness of around 2.5 mm^5^.

CN analysis using NGS data (CNVseq) has allowed researchers to identify structural variation^6,7^, although its limitations remain cost and access to suitable tissue samples. Fresh-frozen primary melanoma tumour tissue with matched normal would be the ideal specimen, but are rarely available in normal clinical practice. The most plentiful source of research material is archived FFPE samples, and therefore for clinical practice and research using tumour data alone would be preferable. However, FFPE tumours yield fragmented DNA, often of low concentration and, for melanoma, the DNA is usually contaminated with the pigment melanin, which inhibits polymerase activity^8^. Low coverage CNVseq has however been successfully used on FFPE lung and oral tumours^9,10^.

Our focus was the characterisation of a large-scale, population-based epidemiological study of incident melanoma. Melanoma is a cancer of major interest with increasing incidence, and extensive mutation profile^11^. Mutation analysis has characterised crucial melanoma oncotargets (e.g *CDKN2A*, *BRAF*, *NRAS*, *TP53*, *CDK4*, *PTEN*, *NOTCH2)*^12^ and this has already made an impact (e.g. BRAF V600E-targeted therapy), but systematic genome-wide CN analysis would permit a broad range of investigations. We report the application of whole-genome CNVseq to small FFPE primary melanomas. We assessed the quality and the discriminatory power of CNVseq at high coverage (1.8x–9.1x) compared to previous studies to detect and distinguish CN changes with a resolution of ≤10 kb (potentially as high as 1 kb). To demonstrate performance, we report the spectrum of genomic CNAs detected at 9p21 and regions involving *PTEN* and *NOTCH2* genes.

## Results

### Sample processing

**Figure 1:**
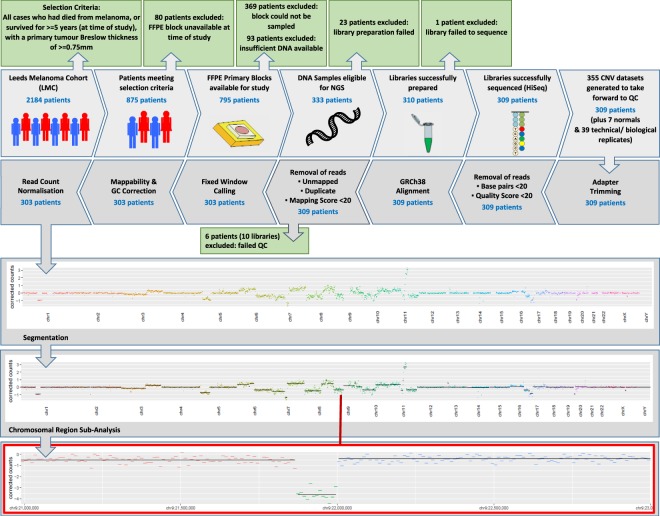
Workflow of samples from tumours sampled from the Leeds Melanoma Cohort. The path below shows how sample numbers are reduced through the process. The Figure also shows the bioinformatics stages through the process. This figure was constructed using SmartDraw 2007 v8.16 Healthcare Edition (www.smartdraw.com).

Figure 1 and Supplementary Fig. 1 detail the workflow and sample attrition. Of the identified 875 participants, 426 tumour blocks were available and suitable for sampling, but 93 (21.8%) did not yield DNA (0 ng DNA mass), leaving 333 blocks for analysis. Libraries were successfully generated for 93.1% (310/333) of these participants (Fig. 1). In terms of predicting successful library preparation, only starting DNA input showed statistical evidence (Wilcoxon rank sum, p < 0.001) (Supplementary Fig. 2). 94.6% of samples with 25–1000 ng of DNA produced libraries, as compared to 72.4% if the input was <25 ng. Melanin score and age of the block did not affect success (Supplementary Fig. 2).

### NGS performance

**Figure 2:**
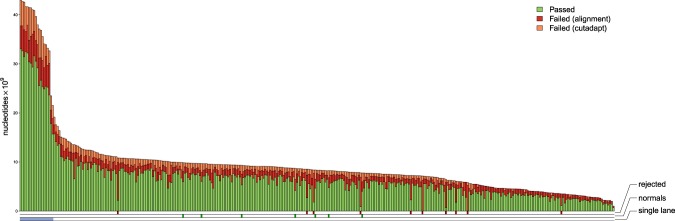
Nucleotide summaries for each library. Overall, for the 335 libraries sequenced at 5 per lane, sequencing yielded an average of 82.1 × 10^6^ reads (21.3 × 10^6^–191.4 × 10^6^). For the 20 libraries sequenced at 1 per lane, there was an average of 368.2 × 10^6^ reads (215.3 × 10^6^–425.6 × 10^6^). The total nucleotide count (across both pairs) for each library is represented by the bar height. This is divided into nucleotides removed during preprocessing (“failed cutadapt” in orange) and nucleotides removed during alignment and read post-processing (“failed alignment” in red). The remaining nucleotides (“passed” in green) are present in the downstream analyses. Library annotations are shown below the plot. The libraries run at a higher depth at one library per lane are marked (“single lane”). The 10 libraries identified as low quality and subsequently rejected are marked (“rejected”), as are the seven libraries from normal samples (“normals”). Following pre-processing (adapter trimming, dropping of reads less than 20 bp, and quality score less than 20), a median of 79.1 × 10^6^ (5.5 × 10^6^–189.3 × 10^6^) reads (multiplexed) and 366.1 × 10^6^ (219.6 × 10^6^–423.8 × 10^6^) reads (single) were successfully aligned. After alignment, deduplication, and read quality filtering, a median of 65.1 × 10^6^ (multiplexed) and 320.0 × 10^6^ (single) reads were retained. 10 libraries which were clear outliers, showing exceptionally low alignment rates (3.1 × 10^6^–34.3 × 10^6^ aligned reads retained), were rejected from subsequent analyses. Overall, the non-rejected libraries gave a median alignment rate of 0.82 (0.47–0.90) yielding a median coverage of 1.8x (multiplexed) and 9.1x (single).

A total of 355 libraries were sequenced (including replicates, and libraries from “normal” tissue). Figure 2 summarizes read count processing. Ten libraries were dropped because of low alignment rates (Fig. 2, Supplementary Fig. 3). All dropped libraries were from the <25 ng group, so that in total for this group, 34.5% of the starting tumours provided meaningful data, while this figure was 94.0% for the samples with 25–1000 ng input DNA. The remaining libraries had a median alignment rate of 0.82 (range: 0.47–0.90) yielding a median coverage of 1.8x (5 libraries multiplexed per lane) and 9.1x (non-multiplexed). Input DNA quantity, melanin content, and age of FFPE block within the range considered here did not meaningfully affect the proportion of aligned reads (Supplementary Fig. 3).

NGS data were successfully generated from primary melanomas for 303 LMC patients (91.0%; 303/333). Characteristics of these participants and the whole LMC can be found in Supplementary Table 1.

For statistical analysis and visualisation of our results, each read is assigned to a specific pre-defined “window” of fixed size based on the alignment coordiates. Larger size windows will include more reads, giving the CN estimate more precision but potentially missing regions of CN change substantially smaller than the window size. Too small a window size and the random nature of this process will hide patterns of CN changes. Under specific assumptions and requirements, calculations can be done to determine the optimum window size^13^. However, in clinical studies such as this, samples are of varying quality and quantity. We chose 10 kb window sizes as being the optimal size for displaying the majority of our sample data, having investigated window sizes ranging from 1 Mb (20 k read per window) to 1 kb (18 reads per window on average) (Supplementary Fig. 4).

### Replicates analysis

We examined different types of paired samples ranging from analysis of cores from 2 separate tumours from the same person, 2 cores from the same tumour and repeat analysis of the sample from the same core. To assess the similarity, we computed a correlation of the adjusted read count in each window across the paired samples. The 3 paired samples resulting from analysis of 2 distinct tumours from each participant did not show a strong correlation (p > 0.05) while the other 35 paired comparisons showed correlations significant at p < 0.05 with the majority (29 out of 35) being significant at p < 0.0001 (Supplementary Fig. 5). We take this to demonstrate that the sample and data processing methods were suitable, and that the input DNA mass was not critical for high-quality data, as long as the input exceeded the 25 ng limit described above.

### *CDKN2A*

To assess the degree of detail in this analysis, we systematically examined the *CDKN2A* region. We conducted segmentation analysis across a feasible space of parameter values (Supplementary Fig. 6). Overall, the results were robust to the precise choice so we took alpha = 0.03 and a standard deviation of 3.

**Figure 3:**
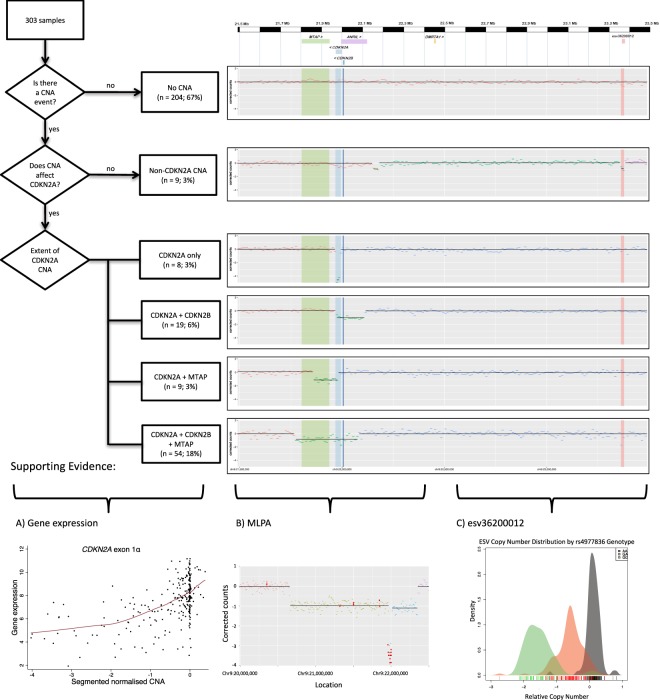
Summary of 303 distinct tumour samples examined for chromosome 9p21 in the *CDKN2A* region (*CDKN2A* (chr9:21,967,753–21,995,301); *MTAP* (chr9:21,802,636–21,865,971); and *CDKN2B* (chr9:22,002,903–22,009,363) showing that 204 of these samples (67%) showed no evidence of a CN change. Among the 99 samples showing a CN change, various patterns are observed, the majority of which impacted in *CDKN2A*, the known target of common alteration for melanoma. Three lines of evidence support the findings from the bioinformatic analysis: (**A**) (bottom left) gene expression analysis of regions of p16 (a protein product of *CDKN2A*) are associated with the extent of deletion in keeping with tumour heterogeneity and subsets of cells having a deletion (see also Supplementary Fig. 11); (**B**) (bottom centre) replication of results in a subset of tumours via MLPA. MLPA mean ratios (red dots) are superimposed (see Supplementary Table 2); and (**C**) evidence that a germline CN variant, esv36200012 (chr9: 23362412–23378071), can be detected reliably. The bottom right panel shows that the measured extent of CN loss matches the expected genotype.

**Figure 4:**
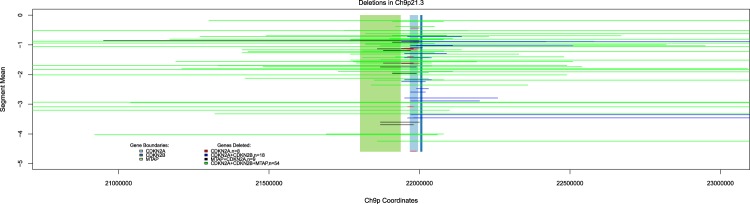
A summary of the regions deleted on 9p21 in the vicinity of *CDKN2A*. The limits of the coding regions of the genes in the region is shown vertically along the chromosome while horizontally the boundaries of the deleted regions produced by the segmentation assay are shown. As expected, the target of the deletions is *CDKN2A*. Gene boundaries: *MTAP* = green; *CDKN2A* = light blue; *CDKN2B* = blue.

Of the 303 individuals samples, 204 (67%) had signal comparable with the average of the genome, suggesting no CN change in the region. 99 of the 303 samples exhibited CN change, with the vast majority (90/303; 29.7%) affecting the *CDKN2A* gene (Fig. 3). CN changes were classified by their size (in nucleotides), and log2 normalised amplitude of the variation (relative to the chromosome value for the sample); and also by their effect on *CDKN2A*, *MTAP*, and *CDKN2B* at 9p21 (Figs 3 and 4). The size of the regions of loss varied from narrow focal 20 kb regions (2 windows) to wide regions extending beyond the 4 Mb region analysed (range = 20 kb–10.63 Mb; median = 380 kb; SD = 2.3 Mb). There was also variation in the amplitude of the loss (log2 normalised amplitude range = −0.18 to −4.58; median = −1.44; SD = 1.03).

Using smaller window sizes of 5 kb and 1 kb, we were able to identify smaller limits of deletion around the *CDKN2A* region. The probable minimum size of deletion that might be detected is 2 kb (2 windows at 1 kb window size). In our data, the minimum region of deletion observed was 5 kb at 1 kb window size (Supplementary Fig. 7).

A number of complex CN patterns were observed (Supplementary Fig. 8). Five samples showed a double loss of the *CDKN2A* region (two separate CN losses affecting the gene). Five samples showed loss at *CDKN2A* plus an additional region of loss elsewhere on 9p21. One sample showed a relative CN gain at the two windows (20 kb) containing *CDKN2A* exon 1β, *CDKN2B*, and *CDKN2B-AS1 (*ANRIL). Nine samples showed a relative CN loss that did not directly affect the coding exons of *CDKN2A*.

### *PTEN* and *NOTCH2*

We reviewed (i) *PTEN* revealing that 16/303 (5.3%) of samples showed a relative CN loss that encompassed the *PTEN* coding region (Supplementary Fig. 9) in keeping with the literature, (ii) while *NOTCH2* fell within a blacklisted region proximal to chromosome 1 centromere and no useful information could be obtained (Supplementary Fig. 10).

### *CDK4* and *MDM2*

We also reviewed *CDK4* and *MDM2* revealing that 16/303 (5.3%) of samples showed a large bipartite amplification of these two genes (Supplementary Fig. 11) in keeping with the literature^14^.

### CNV-Seq validation

To assess the accuracy of the information and patterns gleaned from CNVseq, we conducted three separate examinations:

A) We examined genome-wide gene expression data from these tumours (DASL HT12.4 array) to compare gene expression in *CDKN2A*, *CDKN2B*, *MTAP* and *PTEN*. (Fig. 3A; Supplementary Fig. 12). CN loss at each of these genes was accompanied by a highly significant 2 to 5-fold reduction in expression. We also examined CN gain at *KIT*, a known site of amplification^15^, and showed a significant association between *KIT* amplification and *KIT* expression (Supplementary Fig. 12).
B) As a direct comparison, we conducted MLPA focused on 9p21 in 37 tumours included in CNVseq. While 22 (60%) samples failed to give any results due to DNA fragmentation in FFPE tissue, 13 of the 15 samples that could be analysed were concordant with the CNVSeq in showing heterozygous or homozygous deletion at *CDKN2A* (Supplementary Table 2, Fig. 3B; Supplementary Fig. 13). For the remaining two samples, MLPA indicated *CDKN2A* CN loss not picked up by segmentation with the utilized parameter values.
C) The germline CNV (esv3620012) involving a 16 kb region lies within 9p21. Within the 1000 Genomes Phase 3 dataset, we identified SNPs, including rs4977836, in complete linkage disequilibrium with esv3620012. On the basis of this SNP we predicted the CN for each of our participants. We compared the adjusted read counts in the two 10 kb windows covering esv3620012 with the 10 adjacent windows each side (to remove somatic changes in the region). A 3-peaked distribution was apparent (Fig. 3C; Supplementary Fig. 14) consistent with the imputed genotype at this locus.

## Discussion

The success of TCGA and similar studies in terms of the novel insights gained into mutation profiles and immune responses to cancer is readily apparent. Similarly, CN profiling has contributed to patient stratification and increased understanding of the biological processes driving tumour progression^16,17^. The TCGA dataset however was built by worldwide collaborators who could identify tumours of sufficient size to cryopreserve tissue. Analysis of such highly selected tumours, chosen primarily for availability, size and other *ad hoc* reasons cannot contribute to inferring impact or prevalence in the general population. To make such assessments and to identify drivers most relevant in routine clinical practice requires unselected samples and systematic analysis. To that end, we set out to develop a technique which could be applied in epidemiological analyses of population-based samples and samples stored from mature clinical trial participants; a reproducible, scaleable, efficient technique which produces readily interpretable data. In this instance, the CNVseq results can be combined with cohort data (e.g. survival data, UV exposure patterns), but the ability to generate CN data from FFPE from participants in clinical trials would enable both biomarker discovery and validation required as precision medicine becomes routine. This study reports CN changes in primary melanomas: tiny core biopsies 0.6 mm in diameter, reflecting an extreme technical challenge for cancer genomic studies.

With no proven approach to the systematic analysis of such challenging tissues, we set out to determine if we could modify CNV-seq to produce meaningful results. CN data have historically been generated from melanocytic lesions/cancers using fluorescence *in-situ* hybridization of selected chromosomes^18,19^ and then array-based comparative genomic hybridisation (aCGH)^1,5^. The coverage achieved using these approaches was limited, and NGS has in more recent times been used in research moving towards clinical utility for examination of blood^20^. Given the requirement of routine sampling, approaches to the use of NGS in FFPE samples were made^9^.

Formalin fixation results in the degradation of DNA which is pH dependent^21^. Fixation processes adopted by laboratories vary and therefore the degree to which degradation occurs is thought to vary between laboratories and to increase over time^22^. In this study, blocks were sampled at a median of 9 years. The quality of the CN data at study inception was therefore carefully assessed. We compared detection of the most frequent deletion in melanoma (*CDKN2A*) to data generated using MLPA as a high sensitivity method. Where the DNA was sufficient for PCR, 13/15 MLPA-detected deletions were picked up by our segmentation analysis. Thus the approach was judged to have adequate sensitivy at 9p21, which moreover we showed detected small deletions, the smallest being 5 kb, using a 1 kb window size.

We have shown, for the first time, the evaluation of CNVseq at a relatively high coverage in a large-scale-study using FFPE primary melanomas. We managed to prepare libraries from DNA samples which generated high quality sequencing data where the amount of input DNA was as low as 12.1 ng, but also from a single sample where input DNA mass was below the detection limit of the DNA quantification assay (1 ng). Overall, libraries were made from 78% of 0.6 mm tumour cores extracted. Our aim was to generate data with higher coverage compared to previous CNV-seq studies to detect small CN changes and therefore we tailored our analysis pipeline based on the needs of our dataset. Our analysis pipeline is similar to that described by Scheinin *et al*.^23^, with an additional feature being the normalisation against a pooled “normal” generated from excised normal tissue from similar FFPE tumour blocks. We also excluded regions which have caused spurious CN peaks in previous studies^24^, while Scheinin *et al*. excluded problematic regions identified empirically^23^.

We made the decision to focus on the tumour material and not to analyse matched normal samples concurrently. While this means that we could not immediately distinguish germline and somatic changes because of the lack of a normal comparison tissue sample for each tumour, detailed genetic assessment by imputation of germline CN can be used instead as demonstrated here and employed in similar approaches^25^. This approach would be advantageous where germline DNA would not necessarily be available, for example in analysis of tumour blocks stored from mature clinical trials.

We have shown the reliability of the CNVseq data when using small DNA quantities by looking at the correlation of CN profiles of replicate libraries prepared with different DNA mass. CN changes were associated with gene expression profiles of *CDKN2A*, *CDKN2B*, *PTEN*, *MTAP;* and changes in the 9p21 region were validated using the MLPA technique. The poor quality of the MLPA data, with a 60% fail rate, clearly demonstrates the challenges of using FFPE-derived tissue.

In summary, we have successfully generated CN results for 303 patients using FFPE primary melanoma-derived DNA. The success rate for library preparation and sequencing was 78% cores overall. The application of NGS to formalin fixed tumour samples in this way was shown to produce high sensitivity to CNA at 9p21 and this approach can now therefore be explored in biomarker identification and studies addressed to understanding melanoma biology.

## Materials and Methods

### Participants

The Leeds Melanoma Cohort (LMC) Study^26^ (approvals: MREC (1/3/057), PIAG (3-09(d)/2003)) is comprised of 2184 population ascertained melanoma participants recruited since 2000 and with detailed phenotyping, biological samples, and epidemiological data. Genome-wide SNP data are available from the germline for the majority of participants. Informed consent was obtained from all participants. Case participants were consented to answer questionnaires about their medical history and UV and other exposures, provide a blood sample for genetic and other analysis, allow access to medical records and permit study investigators to access and analyse participants’ pathology material. Processed samples were stored using study-specific identifiers; no person conducting the bioinformatic and statistical analysis presented here had access to any identifiable information.

For this CN assessment, we identified all participants who had died from melanoma when laboratory activities began. As a comparison group, we identified participants who had survived for at least 5 years from diagnosis. Primary tumours from the 875 participants who satisfied these criteria are the focus of this study. These tumours were those obtained from routine clinical practice and were obtained from about 20 distinct pathology laboratories.

Two tumour samples from participants in the Leeds Chemotherapy Study (LCS) (approvals: Yorkshire and Humber Central REC [10/H1313/72] NIGB [ECC 8-02 (FT2)/2010]) with extensive FFPE tumour material were used to assess the effect of differing DNA input amounts. A further 7 sentinel node biopsy (SNB) negative lymph node blocks were sampled as ‘normal’ reference DNA for downstream normalisation of generated sequencing data from formalin fixed tissue.

### Tissue acquisition

Primary FFPE blocks were retrieved from NHS Pathology Departments. Haematoxylin and eosin-stained sections were generated and reviewed by JNB. Sampling was not performed when there would be insufficient tumour remaining for subsequent clinical testing, or if the tissue block lacked useful material (e.g. the tissue was necrotic).

A 0.6 mm diameter tissue microarray needle was used to sample the tumour, horizontally through the deepest part of the tumour which was least contaminated with stroma or inflammation^26^. The degree of pigmentation seen in the histopathology slide was scored for melanin content (from 0–3; absent to heavily pigmented).

### Generation of NGS libraries

DNA was extracted from each tumour core using the Qiagen AllPrep® FFPE kit. DNA samples were quantified using the Quant-iT™ broad range ds-DNA assay kit (Invitrogen™, Life Technologies, USA) according to manufacturer’s recommendations.

Whole-genome DNA libraries were prepared using either a previously described library preparation method^9,27^; or using the NEBNext® Ultra^TM^ DNA Library Prep kit for Illumina® (indexed primers) (New England BioLabs, UK). Automated library preparation using FFPE derived DNA was found to produce libraries of variable quality (data not shown) so all libraries were subsequently prepared manually. We used 5 DNA samples to ascertain that libraries of higher yield were prepared after bovine serum albumin (BSA) addition compared to libraries prepared from a second tumour core from the same samples without BSA addition (data not shown). Library preparation protocols were modified by adding 5 mg/ml BSA as a blocking agent to each reaction to reduce PCR inhibition previously ascribed to melanin^8^.

### Sequencing and alignment

All NGS libraries were sequenced on an Illumina GAII (initial 75 samples) or HiSeq sequencer (all subsequent samples) to produce >100 bp paired-end reads (either 5 or 1 per lane). Sequence reads were trimmed using cutadapt version 1.8.3; adapters and low quality read tails (quality score <20) were trimmed, as were reads <20 nt.

The remaining reads were aligned against the GRCh38 human reference (without alternate contigs) using bwa mem 0.7.10^28^. After alignment, duplicates were marked using Picard (version 1.119) (http://broadinstitute.github.io/picard/). Local realignment was performed to minimise artefacts around known common indels using the GATK pipeline (version 3.4.46) (RealignerTargetCreator and IndelRealigner) using default parameters with the addition of “filter_bases_not_stored” and “filter_mismatching_base_and_quals”^29^. BAM file MD tags were then re-called using samtools (version 1.2) calmd^30^. Trimmed data were finally produced by excluding all alignments that were unmapped, secondary, QC failed, duplicated, or supplementary. In addition, alignments with a mapping quality of less than 20 were excluded.

### Window assignment

The analysis is based on binned counts from identical window locations. Previous studies^23^ have shown that this technique works with window read counts as low as 60^13^. Different fixed window sizes (1M, 100K, 10K, 5K, and 1K basepairs) across the whole genome were explored. Binning was performed using bamwindow (https://github.com/alastair-droop/bamwindow). Reads were assigned to bins using the read midpoint, so that each read fell into exactly one window. Clipped regions were excluded from reads when calculating the midpoint.

### Read count normalisation

Window read counts were normalised to reduce technical variation: the GC content for each window was generated from the reference genome (excluding bases masked as N). Mappability was calculated using the gem-mappability software (version 1.315)^31^ allowing 1 mismatch and a sequence length of 35. This provided a mappability score for each 35-nucleotide sliding window. These fine-scale data were converted to a median mappability score for each window using an in-house Python script (available online from: https://github.com/alastair-droop/windowWIG). LOESS was used to model and adjust for GC content for each chromosome individually and subsequently to adjust for mappability; analyses were conducted using the R routine LOESS using a re-descending M estimator with Tukey’s biweight function (i.e. using family = “symmetric”). A “composite normal” read count for each window was created by summing the raw window counts for the seven SNB-negative ‘normal’ samples. The composite normal was adjusted in the same way as the individual samples. All individual samples were then compared to the corrected composite normal sample to yield a CN estimate.

### Window blacklist generation

NGS read alignment to certain genomic regions is poor; as these regions would be unreliable, we attempted to exclude them. Problematic regions defined previously^24^ as yielding spurious CN peaks were blacklisted. To remove regions with low coverage, windows with zero reads in more than 5% of the samples were blacklisted, individual runs of <150 windows flanked by blacklisted windows were also blacklisted.

### Replicate analysis

Replicate samples were sequenced to assess the reproducibility of our approach. Several types of replicates were assessed: (a) library re-sequencing (“technical”); (b) library generation by different methods using aliquots of the same source DNA (“method”); (c) libraries generated with differing DNA quantities of aliquots of the same source extraction (“concentration”); (d) libraries generated from distinct cores of the same tumour (“core”); (e) libraries generated from two separate primaries from the same patient (“tumour”).

For each replicate type, the pairwise concordance between the replicates was calculated (based on the whole genome) using a Pearson correlation of the read counts after QC and GC and mappability correction and a window size of 10 kb; blacklisted windows and reference genome gaps were excluded from this calculation (“corrected” row in Supplementary Fig. 5). To develop an empirical measure of the significance of the correlation we correlated all pairs of samples where the 2 samples did not come from the same person. This is the null distribution for the correlation (based on the ~45,000 distinct pairs among the 300 persons included in the study) and the significance of replicate samples is estimated from this empirical distribution. For comparison, we show the distribution of correlations prior to adjustment (“raw” in Supplementary Fig. 5) and following segmentation (“segmented” in Supplementary Fig. 5).

### *CDKN2A, PTEN, NOTCH2, CDK4, MDM2* and *KIT* regions

We reviewed the evidence for changes in CN within a 4 Mb region including *CDKN2A* (chr9:21,967,753–21,995,301), the major focus of CN loss in melanoma^32,33^. We also investigated the changes around five other known loci of CNA in melanoma: *NOTCH2*^34^ (chr1: 119,911,553-120,069,626), *PTEN*^35^ (chr10: 87,863,113-87,971,930), *CDK4*^14^ (chr12:57,747,727-57,756,013), *MDM2*^14^ (chr12:68,808,176-68,850,686); and *KIT*^15^ (chr4: 54,657,918-54,740,715).

### Characterisation of segmentation parameters and window size

CN changes were identified using segmentation of the read count data to indicate regions with increased or decreased read counts as compared to the chromosomal average. These could of course represent somatic changes or germline variation.

Log2-normalised read counts were segmented using the DNA copy circular binary algorithm^36^; the code was obtained from https://bioconductor.org/packages/release/bioc/html/DNAcopy.html; analysis reported here was completed with version 1.56. Default values of parameters were accepted for the analysis except for 2 parameters: alpha which is the significance level at which a putative break-point is accepted and the standard deviation which is the minimum limit for an accepted break point to be retained. The values of these two parameters were determined using a state-space exploration focused on the *CDKN2A* region with a window size of 10 kb. For each point in the state-space, the calculated segments were compared to a reference set. We covered alpha values from 0.0005 to 0.05, but not evenly. We covered SD values from 0.1 to 0.5 in increments of 0.05. This reference set was generated from: knowledge of the region (loss at *CDKN2A* has been consistently reported in around 30% of melanomas^32,33^); comparison of replicates within the data set; and an evaluation of each potential CN change at multiple stringencies.

In order to assess the effect of window size on the CN analysis, the *CDKN2A* region was evaluated using 10 kb, 5 kb and 1 kb bp window sizes.

The proportion of melanoma tumours with CN loss observed at 9p21 with the above segmentation parameters was compared to the previously reported frequency (The Cancer Genome Atlas: TCGA). The same parameters were applied to the analysis of *PTEN* and *NOTCH2* to assess applicability for other genomic regions.

### MLPA validation at 9p21 region

Multiplex Ligation-dependent Probe Amplification (MLPA) was carried out on a subset of samples in order to validate NGS CN findings. A total of 37 samples, where sufficient FFPE derived tumour DNA was available, were selected for MLPA analysis. The mass of the input DNA used for the experiment was 50–100 ng. 19 samples with identified CN loss at the *CDKN2A* region based on results of the NGS analysis, and 18 samples with no detected loss at the *CDKN2A* region were analysed. DNA extracted from blood, FFPE tonsil, and FFPE lymph node were used as reference samples.

MLPA of 9p21 was carried out using the ME024 probemix, which contains 23 probes for the *CDKN2A/2B* gene region (MRC-Holland, Amsterdam, The Netherlands). Hybridisation, ligation, amplification, and PCR, were carried out according to the MLPA two-tube protocol (MRC-Holland, Amsterdam, the Netherlands). Capillary electrophoresis was performed using Applied Biosystems 3130xl Genetic Analyzer (Life Technologies). Data were analysed using Coffalyser.Net (version v.140721.1958, www.coffalyser.wordpress.com).

### Gene expression analysis

Whole genome transcriptomic data were available for 266 of the tumours sequenced. The RNA samples for this analysis came from adjacent or the same core and the expression profiles were generated using the Illumina whole genome DASL HT12.4 array. Data pre-processing has been described elsewhere and included background correction, quantile normalisation and batch adjustment^37^. We selected a region of chromosome 9p21 containing *CDKN2A (*coding for p16, p14ARF and ANRIL*)*, *MTAP*, *CDKN2B* and a region of chromosome 10 containing *PTEN* to test the correlation between expression levels of these genes and CN changes and thus validate the NGS experiment. Boxplots of gene expression were drawn by CN call (deletion vs. no change) and the difference between groups was tested using the Mann-Whitney U test.

## Supplementary information

Supplementary Information

## Data Availability

The datasets generated during and/or analysed during the current study are available from the corresponding author on reasonable request.
