# Supplementary material for: High-Resolution Copy Number Patterns From Clinically Relevant FFPE Material
Source: Sci Rep. 2019 Jun 20;9:8908. doi: 10.1038/s41598-019-45210-2 (PMC6586881; doi:10.1038/s41598-019-45210-2)
Supplement: Supplementary file 1 — Supplementary Information [file 41598_2019_45210_MOESM1_ESM.pdf]

Supplementary Information

High-Resolution Copy Number Patterns From Clinically Relevant FFPE Material

Anastasia Filia[1,2]^, Alastair Droop[3]^, Mark Harland[1], Helene Thygesen[1], Juliette Randerson-Moor[1], Helen Snowden[1], Claire Taylor[1], Joey Mark S. Diaz[1], Joanna Pozniak[1], Jérémie Nsengimana[1], Jon Laye[1], Julia A Newton-Bishop[1], D Timothy Bishop[1]

^ These authors contributed equally to the work.

[1] Section of Epidemiology and Biostatistics, Leeds Institute of Medical Research at St James's, University of Leeds, United Kingdom

[2] Centre for Translational Research, Biomedical Research Foundation of the Academy of Athens (BRFAA), Athens, Greece

[3] MRC Medical Bioinformatics Centre, Leeds Institute of Data Analytics, University of Leeds, United Kingdom

Supplementary Figure 1: Attrition of participants at each stage of processing. 875 patients met the selection criteria for this study, these were: *cases who had died from melanoma; or survived for >=5 years (at time of study), with a primary melanoma Breslow thickness of >= 0.75mm (see Methods). Of the 875 participants identified, 80 (9.1%) of the blocks could not be located within the NHS Pathology Laboratory. Of the 795 retrieved blocks, 369 (46.4%) were deemed to be unacceptable for sampling without risking compromising the block for future diagnostic use. Of the 426 blocks available for sampling, 93 (21.8%) did not yield sufficient DNA mass for library preparation. Thus, DNA from 333 blocks (78.2%) was available for library preparation, divided into 126 who had died of melanoma and 207 who had survived at least 5 years from diagnosis.

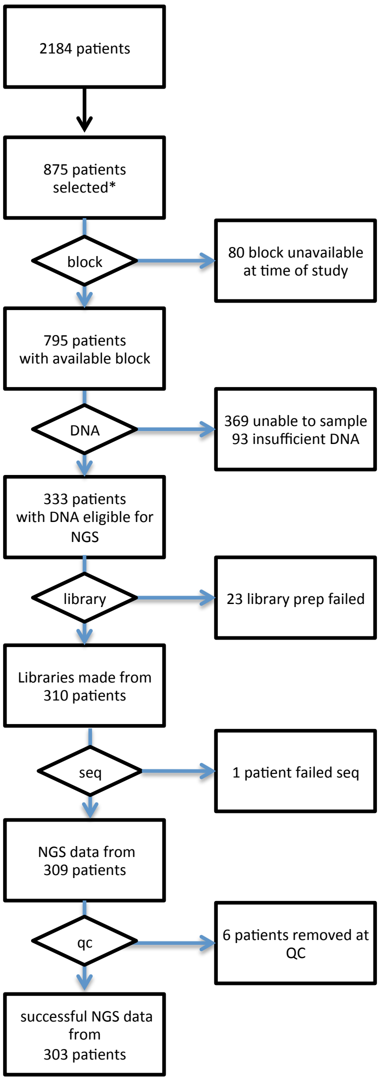

Supplementary Figure 2: Effect of parameters on success of CN analysis. A) input DNA mass for library preparation (in nanograms) showing significant differences between the input DNA quantity in successfully generated libraries and unsuccessful samples ($p = 7x10^{-9}$); B) Tumour melanin content score, using a scale of 0-3 (0 = no melanin present, 3 = high melanin content) finding no differences between the two groups (p=0.46), and C) Age of tumour block, using year of tumour resection (p=0.86).

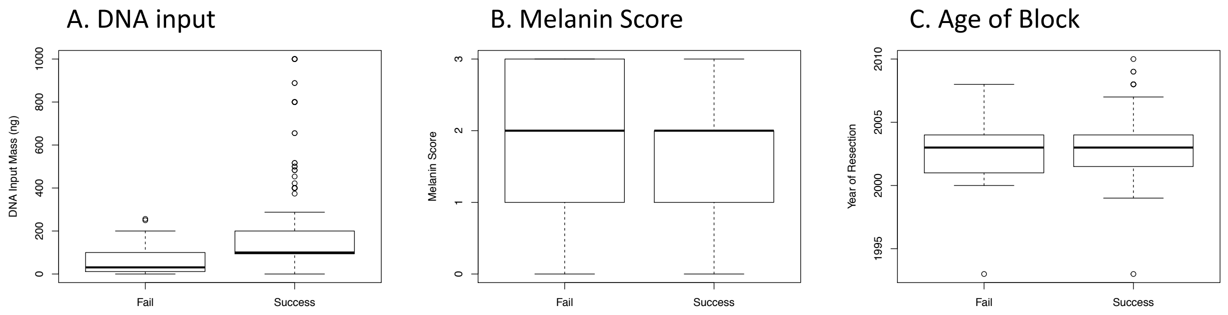

Supplementary Figure 3: Effect of parameters on percentage of reads mapped. A) Input DNA mass for library preparation (in nanograms) indicating improved alignment with increasing DNA input ($p=7x10^{-13}$), but even from the lowest yield more than 75% of reads were aligned; B) Tumour melanin content score, using a scale of 0-3 (0 = no melanin present, 3 = high melanin content). There is no association between proportion of aligned reads with melanin score (p=0.24); C) Age of tumour block, using year of tumour resection. We observed a modest, non-significant decrease in the proportion of aligned reads with age of tumour block (p=0.13); we have no explanation for this counterintuitive trend. Regression lines are shown in solid black. A cutoff of 45% aligned reads (indicated by dotted blue line) was used for QC of sequencing libraries. All libraries with less than 45% aligned reads (indicated in blue) were marked as fails and did not contribute to subsequent analyses.

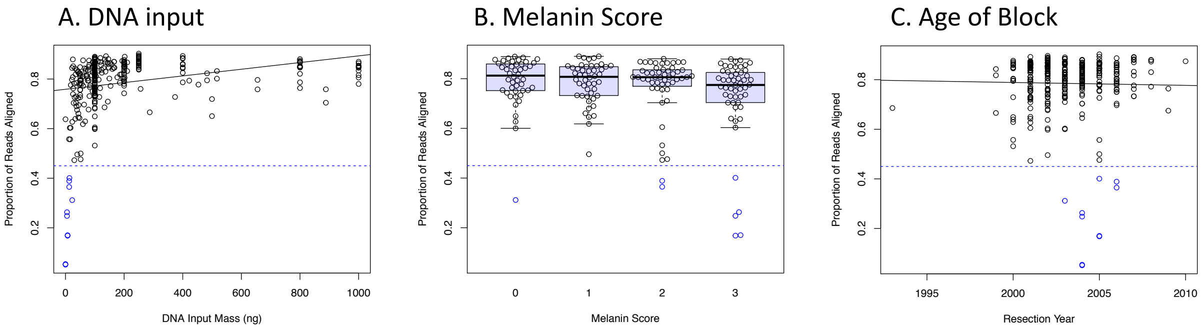

Supplementary Figure 4: Effect of window size on segmentation efficiency at 9p21. The complete dataset was sampled at 5 window sizes from 1Mb down to 1kb. Larger window sizes provide a greater statistical reliability to CN changes, at the expense of lower resolution. For each window size, corrected data were segmented as described in the materials and methods. The parameters used for segmentation were trained on the 10kb data, so this is expected to yield the best segmentation quality. As can be seen, the 1Mb and 100kb window sizes are too large to reliably determine the copy number variation in this sample. The 1kb dataset, however, is far too noisy for the segmentation to work. In this case, both 10kb and 5kb window sizes show high quality segmentation. Gene boundaries: *MTAP* = green; *CDKN2A* = light blue; *CDKN2B* = blue.

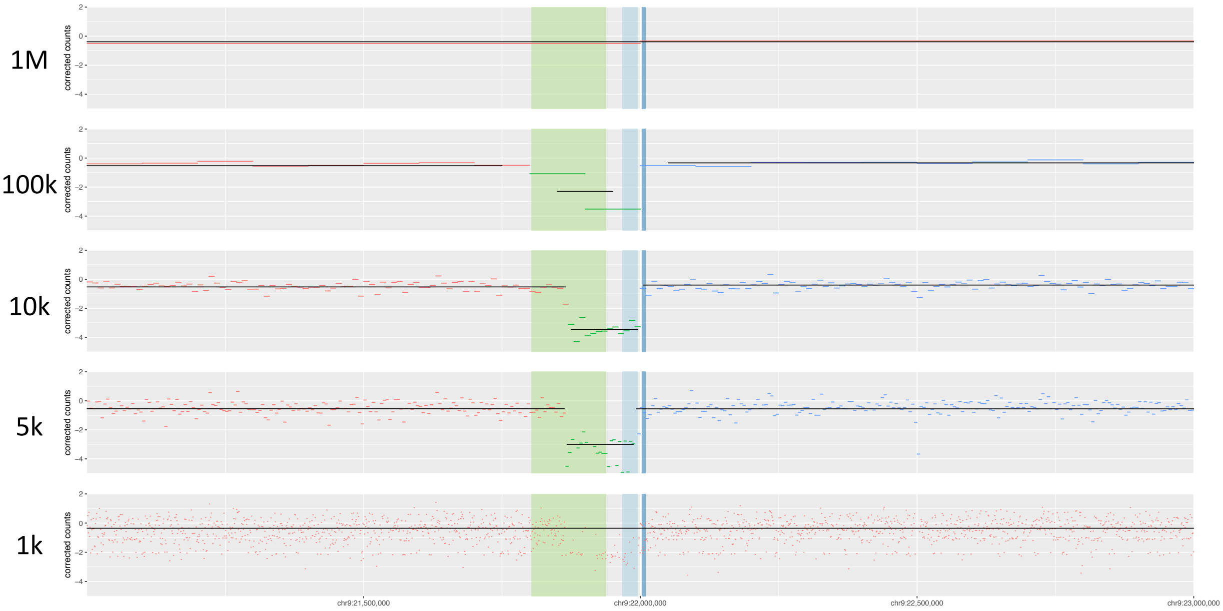

Supplementary Figure 5: Whole genome replicate sample analysis. A total of 38 pairwise comparisons of replicates could be analysed: a) 14 libraries were directly re-sequenced ("technical"); b) 2 libraries were prepared using the different library preparation methods from each of 5 cores (one each from 5 distinct patients) ("method"); c) 5 libraries which were generated using different DNA input from 2 (LCS) patients (3 libraries using 250ng, 100ng and 25ng DNA from patient 1; 2 libraries using 100ng and 25ng DNA from patient 2) ("concentration"); d) 22 libraries derived from 11 patients where a second library was prepared using a second core from the same primary tumour block ("core"); e) 5 libraries derived from different primary tumours from the same patient (3 tumours from one patient, two tumours from a second patient) ("tumour"). The complete (all samples against all) Pearson correlation space was calculated across the complete genome using a window size of 10kb. Individual replicate sample pair correlations were calculated, and superimposed against the background distribution. Replicate pairs are shown grouped by type. A) shows the empirical Pearson correlation distribution for raw read counts. Significant correlation values are marked at 0.05 (*) , 0.01 (**), 0.001 (***) and 0.0001 (****). The dotted line marks the median correlation. B) shows the individual replicate pairs grouped by replicate type. C) shows the numbers of replicate pairs at each significance level. All the technical replicates show extremely good correlation for the raw data, which is improved by both data correction and segmentation. The biological core replicates also show good correlation that increase with processing. The tumour replicates have lower correlation scores than the other replicate types. This is expected, as these samples are derived from different tumours.

A

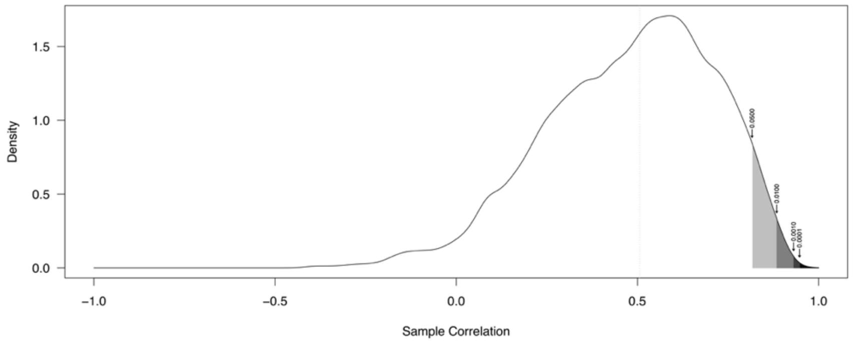

B

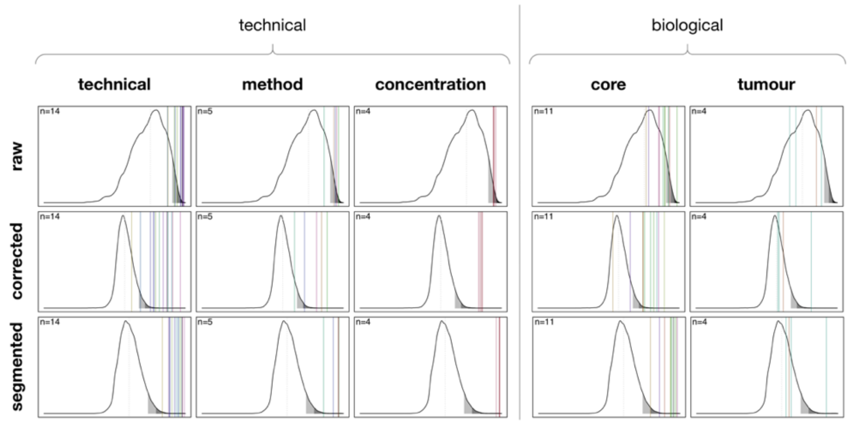

C

		technical	method	concentration	core	tumour
		technical			biological	
raw	–	2 / 14	1 / 5	0 / 4	7 / 11	4 / 4
raw	*	3 / 14	1 / 5	0 / 4	3 / 11	0 / 4
raw	* *	1 / 14	2 / 5	4 / 4	0 / 11	0 / 4
raw	* * *	1 / 14	1 / 5	0 / 4	0 / 11	0 / 4
raw	* * * *	7 / 14	0 / 5	0 / 4	1 / 11	0 / 4
corrected	–	1 / 14	1 / 5	0 / 4	2 / 11	3 / 4
corrected	*	1 / 14	0 / 5	0 / 4	0 / 11	0 / 4
corrected	* *	1 / 14	1 / 5	0 / 4	4 / 11	0 / 4
corrected	* * *	3 / 14	0 / 5	0 / 4	0 / 11	0 / 4
corrected	* * * *	8 / 14	3 / 5	4 / 4	5 / 11	1 / 4
segmented	–	0 / 14	0 / 5	0 / 4	0 / 11	3 / 4
segmented	*	0 / 14	0 / 5	0 / 4	1 / 11	0 / 4
segmented	* *	1 / 14	0 / 5	0 / 4	0 / 11	0 / 4
segmented	* * *	2 / 14	1 / 5	0 / 4	1 / 11	0 / 4
segmented	* * * *	11 / 14	4 / 5	4 / 4	9 / 11	1 / 4

Supplementary Figure 6: Extensive evaluation of the statespace for the CDS parameters (alpha and SD) showing the pattern of errors measured as the difference between the inferred number of fragments detected by the CBS algorithm and the evaluation by an informed reviewer (MH) who scored the pattern across 9p21 blinded to the results of the CBS algorithm. The region of minimal error (the darkest green region in the contour plot) covers a broad range of value pairs, demonstrating a relatively low sensitivity to changes in parameters in this region. We chose SD = 3 and alpha = 0.03 as being representative of this minimal region.

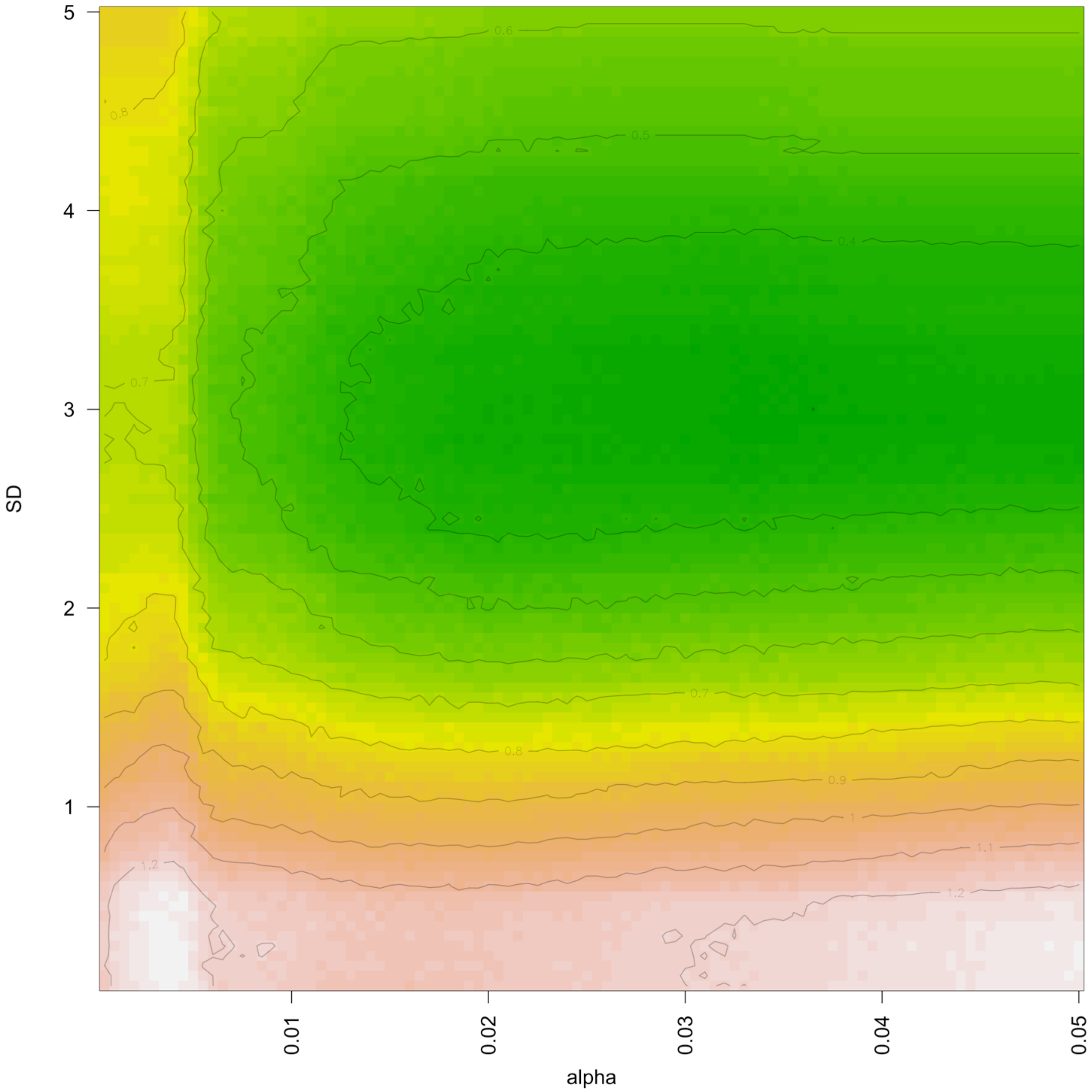

Supplementary Figure 7: The minimum region of deletion observed at *CDKN2A* in the FFPE melanoma samples was a 5kb deletion identified by segmentation at 1kb window size. Gene boundaries: *MTAP* = green; *CDKN2A* = light blue; *CDKN2B* = blue.

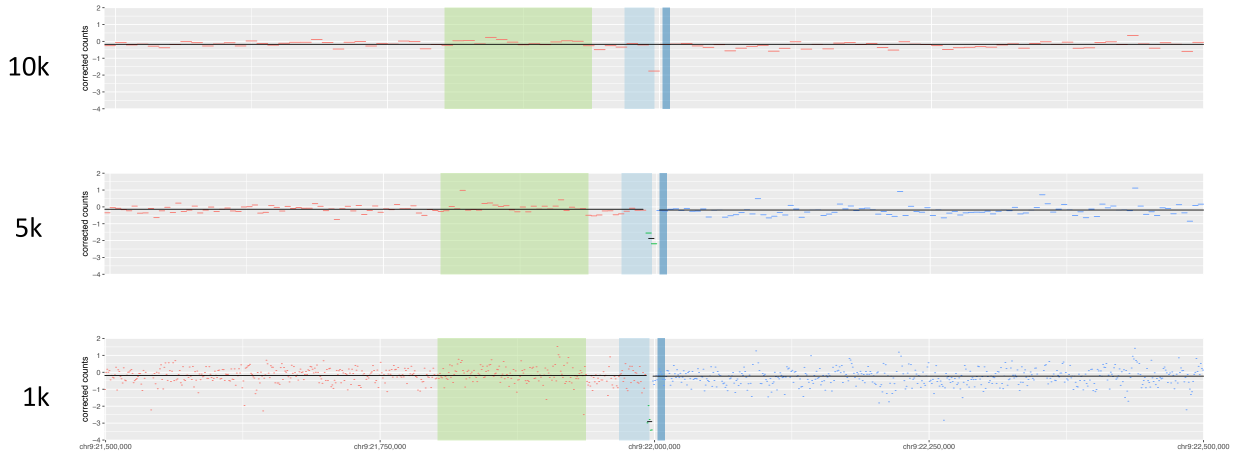

Supplementary Figure 8: Examples of patterns of copy number changes in the *CDKN2A* region but not fitting directly into the patterns shown in Figure 2. Examples include those showing multiple regions of change. **a**) Five of the 89 samples with loss at *CDKN2A* showed a double loss of the *CDKN2A* region, with what appeared to be two separate copy number losses affecting the gene. **b**) Five samples showed loss at *CDKN2A* plus an additional region of loss elsewhere on 9p21. **c**) One sample showed a relative copy number gain at the two windows (20kb) containing *CDKN2A* exon 1β, *CDKN2B*, and *CDKN2B-AS1 (ANRIL)*. **d**) Nine showed a relative copy number change that did not directly affect the coding exons of *CDKN2A* (non-*CDKN2A* CN change). Gene boundaries: *MTAP* = green; *CDKN2A* = light blue; *CDKN2B* = blue.

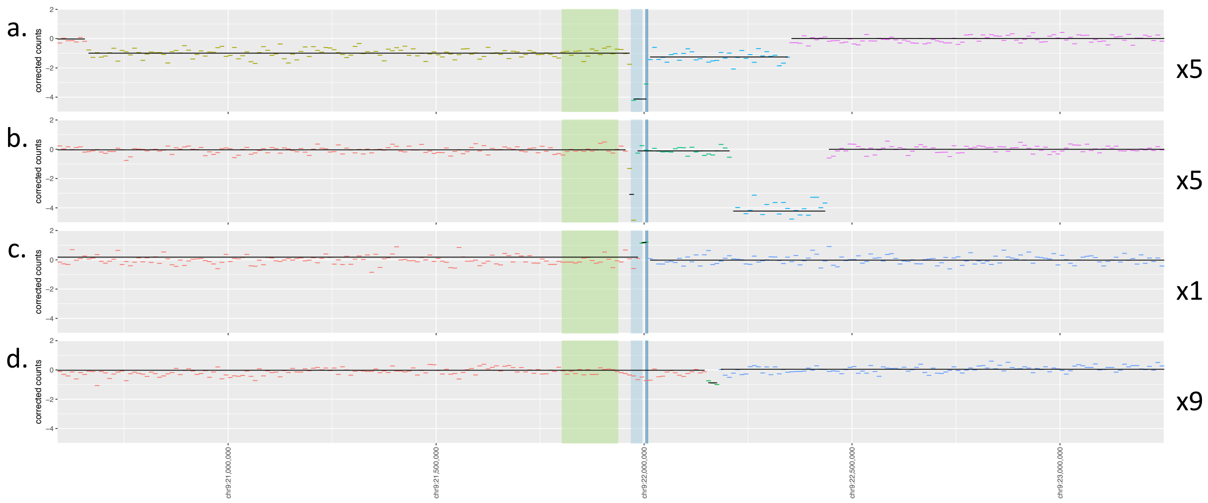

Supplementary Figure 9: Example of copy number loss at *PTEN* (gene boundaries indicated in purple). A large region of copy number loss can be seen extending across the *PTEN* gene. Similar patterns of loss of this region were observed in 16 melanoma tumours.

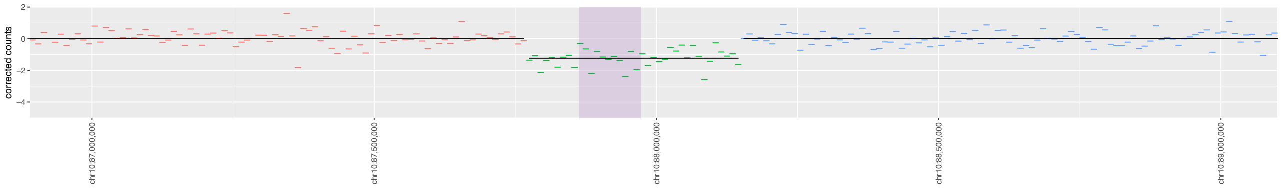

Supplementary Figure 10: Example of poor copy number data at *NOTCH2* (gene boundaries indicated in orange). Due to its proximity to the chromosome 1 centromere a portion of the *NOTCH2* gene falls within a blacklisted region (indicated by brown shading) and cannot be analysed for copy number changes.

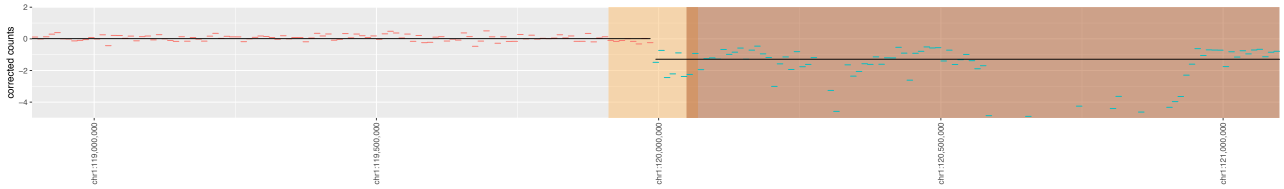

Supplementary Figure 11: Distribution of transcript levels for *CDKN2A* (p16 and p14ARF)*, CDKN2B, MTAP* and *PTEN* by CN change. Profiles of 3 probes are shown for each of *CDKN2A* and *CDKN2B.*

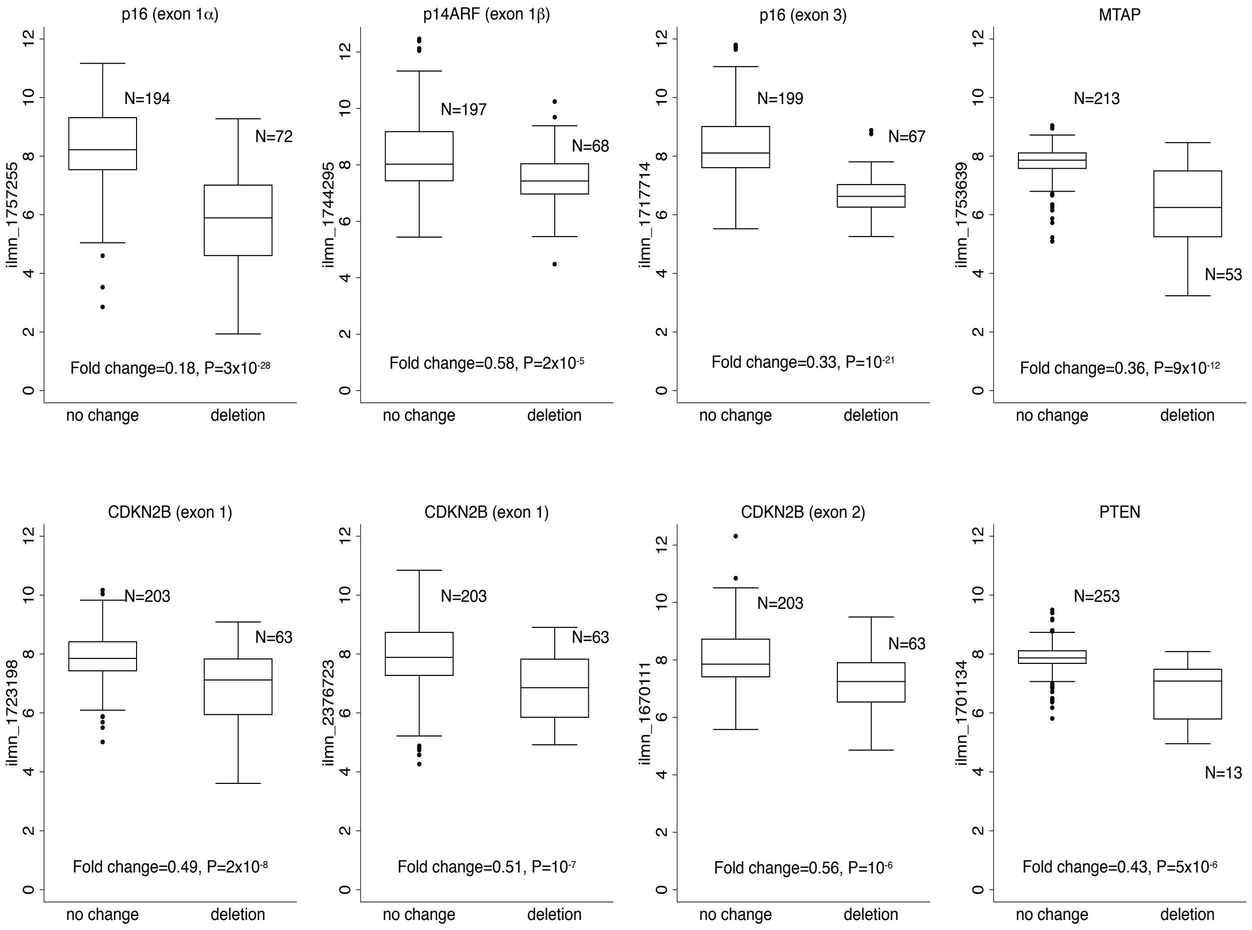

Supplementary Figure 12: Comparison of segmented data to MLPA for chr9p21. In this example, segmented data at a window size of 10kb is shown with log2-normalised MLPA mean ratios superimposed. No scaling has been performed. MLPA probe ratios (red dots) are shown as mean±sd. Gene boundaries: *MTAP* = green; *CDKN2A* = light blue; *CDKN2B* = blue.

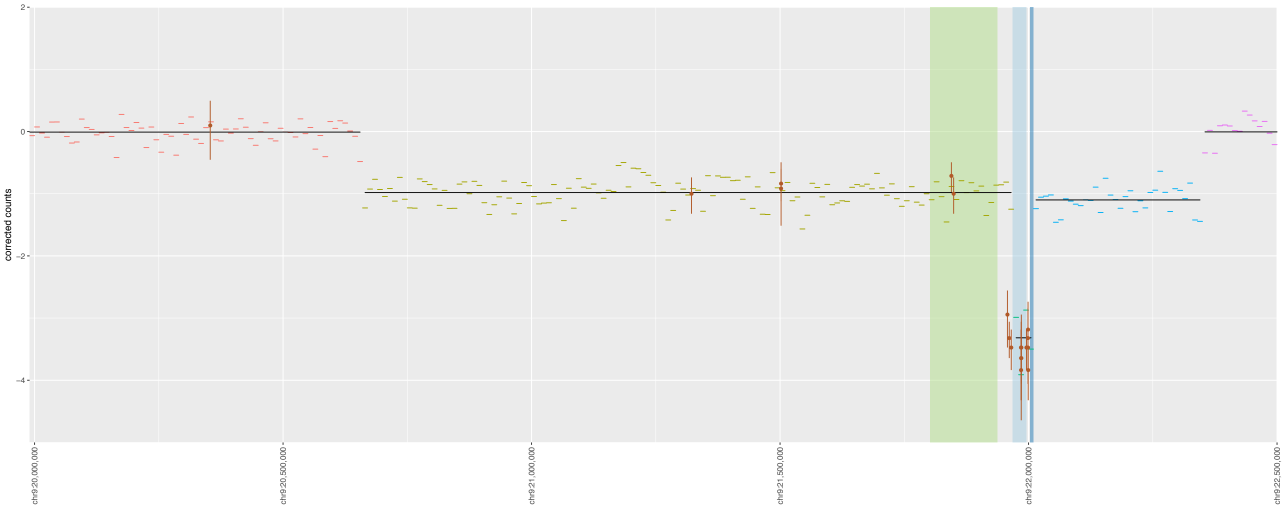

Supplementary Figure 13: Examination of identified common variation in 9p21, postulated to be esv36200012. The distribution of relative copy number aligned to the germline esv (adjusted against the adjacent 10 windows each on the left and right) varies with rs4977836 genotype.

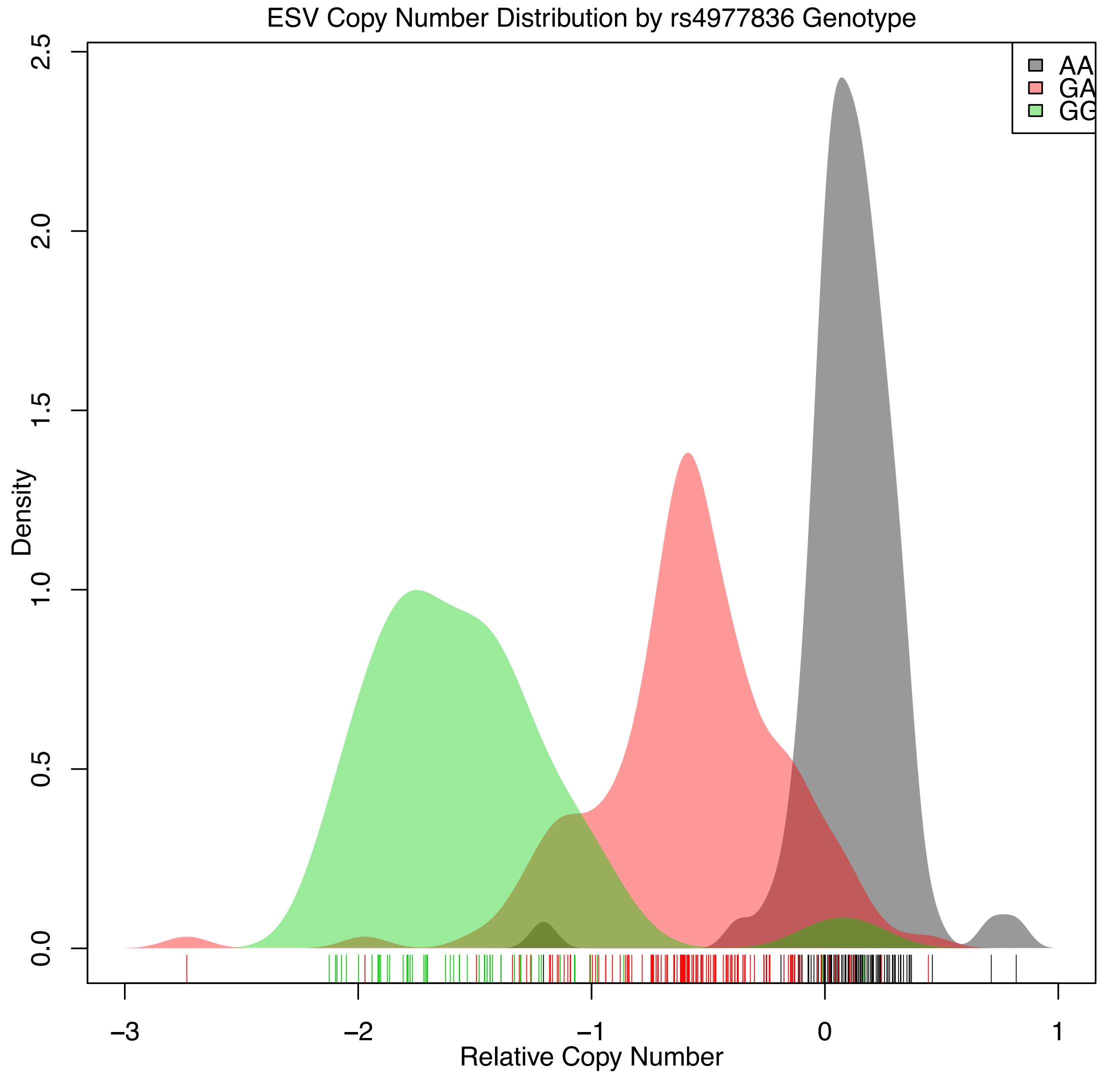

Supplementary Table 1: Characteristics of the 303 successfully analysed Leeds Melanoma Cohort patients and their primary tumours compared to the entire Leeds Melanoma Cohort. In the two patients where a second primary or a metastasis were also examined only characteristics of the first primary are considered for comparisons of Breslow thickness, site of primary and ulceration status.

	**Leeds Melanoma Cohort**	**Successfully analysed CN patient subset**	
		**Survived >= 5years**	**Died from melanoma**
**Samples**	2184	186	117
**Age (median, IQR)**	56 (21)	55 (18)	60 (17)
**Gender (%)**			
Female	1233 (56.5)	104 (55.9)	57 (48.7)
Male	951 (43.5)	82 (44.1)	60 (51.3)
**Breslow (median, IQR)**	1.5 (1.5)	2.26 (1.7)	3 (3.1)
**Site of primary (%)**			
Head/Neck	211 (9.7)	31 (16.7)	15 (12.8)
Trunk	766 (35.1)	53 (28.5)	39 (33.3)
Limbs	959 (43.9)	87 (46.8)	43 (36.8)
Other	248 (11.4)	15 (8.1)	20 (17.1)
**Ulceration**† (%)			
No	1701 (80.0)	131 (70.4)	57 (48.7)
Yes	426 (20.0)	55 (39.6)	60 (51.3)

† Cases were assumed to have no ulceration in instances where ulceration was not recorded. Ulceration status not available for 57 non-primary melanoma cases.

Supplementary Table 2: Comparison of NGS and MLPA copy number analysis. An additional 11 samples failed MLPA.

ID	NGS Result	MLPA Result	MLPA quality	Match
1	double loss at *CDKN2A*	heterozygous and homozygous loss at *CDKN2A*	75%	yes
2	CN loss at *CDKN2A*	heterozygous loss at CDKN2A	10%	yes
3	CN loss at *CDKN2A*	heterozygous loss at CDKN2A	0%	yes
4	CN loss at *CDKN2A*	heterozygous loss at CDKN2A	0%	yes
5	no CN loss	no CN loss	85%	yes
6	no CN loss	no CN loss	70%	yes
7	no CN loss	no CN loss	60%	yes
8	no CN loss	no CN loss	50%	yes
9	no CN loss	no CN loss	50%	yes
10	no CN loss	no CN loss	50%	yes
11	no CN loss	no CN loss	40%	yes
12	no CN loss	no CN loss	20%	yes
13	no CN loss	no CN loss	20%	yes
14	CN loss at *CDKN2A*	not interpretable	30%	n/a
15	CN loss at *CDKN2A*	not interpretable	20%	n/a
16	CN loss at *CDKN2A*	not interpretable	0%	n/a
18	CN loss at *CDKN2A*	not interpretable	-10%	n/a
17	no CN loss	not interpretable	0%	n/a
19	no CN loss	not interpretable	75%	n/a
20	no CN loss	not interpretable	60%	n/a
21	no CN loss	not interpretable	50%	n/a
22	no CN loss	not interpretable	20%	n/a
23	no CN loss	not interpretable	0%	n/a
24	no CN loss	not interpretable	-10%	n/a
25	no CN loss	heterozygous loss at CDKN2A	60%	no
26	no CN loss	heterozygous loss at CDKN2A	0%	no
